# Improving the stability of bivariate correlations using informative Bayesian priors: a Monte Carlo simulation study

**DOI:** 10.3389/fpsyg.2023.1253452

**Published:** 2023-09-07

**Authors:** Carl Delfin

**Affiliations:** ^1^Lund Clinical Research on Externalizing and Developmental Psychopathology (LU-CRED), Child and Adolescent Psychiatry, Department of Clinical Sciences Lund, Lund University, Lund, Sweden; ^2^Centre for Ethics, Law and Mental Health (CELAM), Department of Psychiatry and Neurochemistry, Institute of Neuroscience and Physiology, Sahlgrenska Academy, University of Gothenburg, Gothenburg, Sweden

**Keywords:** Bayesian statistics, sample size, correlation, prior distribution, Monte Carlo simulation, replication crisis

## Abstract

**Objective:**

Much of psychological research has suffered from small sample sizes and low statistical power, resulting in unstable parameter estimates. The Bayesian approach offers a promising solution by incorporating prior knowledge into statistical models, which may lead to improved stability compared to a frequentist approach.

**Methods:**

Simulated data from four populations with known bivariate correlations ( ρ = 0.1, 0.2, 0.3, 0.4) was used to estimate the sample correlation as samples were sequentially added from the population, from *n* = 10 to *n* = 500. The impact of three different, subjectively defined prior distributions (weakly, moderately, and highly informative) was investigated and compared to a frequentist model.

**Results:**

The results show that bivariate correlation estimates are unstable, and that the risk of obtaining an estimate that is exaggerated or in the wrong direction is relatively high, for sample sizes for below 100, and considerably so for sample sizes below 50. However, this instability can be constrained by informative Bayesian priors.

**Conclusion:**

Informative Bayesian priors have the potential to significantly reduce sample size requirements and help ensure that obtained estimates are in line with realistic expectations. The combined stabilizing and regularizing effect of a weakly informative prior is particularly useful when conducting research with small samples. The impact of more informative Bayesian priors depends on one’s threshold for probability and whether one’s goal is to obtain an estimate merely in the correct direction, or to obtain a high precision estimate whose associated interval falls within a narrow range. Implications for sample size requirements and directions for future research are discussed.

## 1. Introduction

It is well known that many findings in psychological research are not replicable, due in large to small sample sizes and insufficient statistical power (Maxwell et al., 2015; Anderson and Maxwell, 2017; Szucs and Ioannidis, 2017; Tackett et al., 2019; Nosek et al., 2022). A large survey of over twelve thousand estimated effect sizes from the psychological literature found that only 8% of the included studies were adequately powered (Stanley et al., 2018), and there has been little to no apparent increase in statistical power during the last six decades, despite a continuous flow of publications emphasizing the importance of adequate power (Sedlmeier and Gigerenzer, 1989; Rossi, 1990; Vankov et al., 2014; Smaldino and McElreath, 2016).

Low-powered studies and small sample sizes pose several challenges. For one, they are less likely to detect a true effect, resulting in an increased rate of false negatives. When true effects *are* detected, the effect sizes tend to be exaggerated, and a statistically significant finding in a low-powered study is more likely to be a false positive than a statistically significant finding in a high-powered study (Fraley and Vazire, 2014; Brysbaert, 2019). Furthermore, small sample sizes result in unstable estimates that rapidly fluctuate in magnitude and even direction as additional samples are added. This notorious instability has been referred to as the “sea of chaos” (Lakens and Evers, 2014), and can result in findings that while statistically significant are in fact in the wrong direction (Gelman and Carlin, 2014; Klein et al., 2018). This “chaos” is not just associated with parameter estimates; others have observed similar properties of *p*-values, though labeling it “fickleness” rather than “chaos” (Halsey et al., 2015; Halsey, 2019).

A relevant question, then, is when does chaos end and stability begin? In the case of bivariate correlations, Schönbrodt and Perugini (2013) sought to answer just that using Monte Carlo simulations. The authors simulated a bivariate Gaussian distribution of *N* = 1,000,000 and a specified population correlation, ρ , then drew 100,000 bootstrap samples, each of *n* = 1,000. For every bootstrap sample they calculated the sample correlation, $\hat{\rho }$ , from *n* = 20 to *n* = 1,000, adding a single observation at each step. This procedure makes it possible to follow the “trajectory” of $\hat{\rho }$ as the sample size increases. The authors defined a Corridor of Stability (COS) around ρ within which all estimated sample correlations are deemed acceptable, based on an effect size measure, Cohen’s *q*, that only depends on sample size (e.g., Rosnow and Rosenthal, 2003). Using their method, chaos ends at the Point of Stability (POS): The sample size at which the trajectory of the sample correlation does not leave the COS. The authors examined different values of ρ and different widths of the COS and concluded that in typical circumstances a reasonable trade-off between accuracy and confidence is achieved when the sample size approaches 250. They also note that there are few occasions where it is justifiable to go below *n* = 150.

For various reasons it may not always be feasible to recruit 250 or even 150 participants (Finkel et al., 2017). The target population could be small or difficult to access, such as in forensic settings (Dumas-Mallet et al., 2017; Pedersen et al., 2021), or the phenomenon might be expensive to measure, such as in neuroscientific research (Mar et al., 2013). A promising solution to the challenge of small sample research, and one that has steadily gained traction in psychological research (Andrews and Baguley, 2013), is the Bayesian approach. Among the more attractive benefits of this approach is that Bayesian hypothesis testing allows researchers quantify evidence in favor of *both* the null hypothesis and any alternative hypothesis. Moreover, Bayesian parameter estimation allows researchers to make genuine probabilistic statements about parameter estimates that are not conditioned on hypothetical future replications, as is the case in frequentist estimation. As such, a Bayesian approach makes it possible to avoid many of the issues stemming from the routine use of *p*-values with arbitrary cutoffs (Wasserstein and Lazar, 2016; Wasserstein et al., 2019). For a more in-depth introduction to Bayesian statistics in the context of psychological research, see Wagenmakers et al. (2018).

Furthermore, Bayesian estimation in small samples has several advantages. One major advantage is that unlike maximum likelihood estimation, Bayesian estimation does not assume large samples, and therefore a Bayesian model should result in estimates comparable to a maximum likelihood model but using less data (Hox et al., 2012; van de Schoot et al., 2015). Moreover, each parameter in a Bayesian model is assigned a prior distribution that is generally chosen so that impossible values cannot occur (Hox, 2020). The prior can also incorporate knowledge from previous research or from expert judgment. For instance, if previous research suggests that the bivariate correlation between scores on two personality measures should be positive, a prior can be constructed that gives more credibility to positive rather than negative estimates. Such a prior should, in theory, decrease the risk of reporting an estimate that is in the wrong direction; committing a *Type S* (for sign) error.

A prior can also be constructed that gives less credibility to extreme values, which should increase precision further and decrease the risk of reporting exaggerated estimates; committing a *Type M* (for magnitude) error (Gelman and Carlin, 2014). Another advantage is that the sample size does not have to be determined *a priori*. With the Bayesian approach, one can simply keep adding samples until a desired threshold is reached, without having to worry about complicated *p*-value adjustments (Rouder, 2014). Finally, the Bayesian approach also provides a more intuitive framework for interpreting statistical results. Rather than relying on *p*-values and null-hypothesis significance tests, Bayesian models produce a posterior distribution that can be directly interpreted as the degree of belief in the hypothesis of interest.

Since the prior influences the estimate, especially at small sample sizes, the choice of prior remains a contentious issue (Stefan et al., 2020). While the use of informative Bayesian priors can outperform frequentist approaches in terms of model accuracy and power, *naively* using a Bayesian approach can lead to worse performance (Smid et al., 2020; Zitzmann et al., 2021a,b). Ideally, prior elicitation and selection should be seen as any other aspect of the research process: It should be backed by theory, well described, and justified in the context of the research question (Baldwin and Fellingham, 2013; Smid et al., 2020). As Stefan et al. (2020) notes, however, there have been few efforts at prior elicitation in psychological research to date, and it is difficult to know *a priori* how different priors will affect one’s parameter estimates. Knowing how much a specific prior influence the sample size required to, for instance, obtain an estimate in the correct direction or reach a certain threshold for stability is especially valuable in research contexts where recruitment is expensive or otherwise challenging.

Simulation-based research can aid prior elicitation by examining how much impact various priors have on parameter estimates, thus providing some initial guidelines for choosing a suitable Bayesian prior. As an example, previous simulation work focused on multilevel models has shown that informative Bayesian priors can produce more accurate estimates compared to a maximum likelihood-based approach, particularly under problematic conditions, and that Bayesian estimates are highly dependent on the choice of prior distribution (Zitzmann et al., 2015). The current study will build upon the work by Schönbrodt and Perugini (2013) and examine the impact of a Bayesian statistical approach to bivariate correlations. Specifically, the current study will investigate the sample size required to conclude, with different degrees of confidence, for different values of ρ , and using different Bayesian priors, that:

1. An estimate is in the correct direction
2. An estimate is robustly different from zero
3. An estimate is within an acceptable range

The first aim relates to the risk of committing a Type S error, and since only positive values of ρ will be used in the current study it is defined as the sample size at which a specific proportion of estimates are above zero. The second aim is related to the traditional notion of statistical power and is defined as the sample size at which a specific proportion of the lower bound of the associated 66, 90% or 95% interval is above zero. The third aim concerns the precision of obtained estimates and the risk of committing a Type M error and is defined here in two ways. First, as the sample size at which a specific proportion of estimates fall inside the COS, and second, as the sample size at which a specific proportion of the associated interval falls inside the COS. It should be noted that these definitions differ slightly from Schönbrodt and Perugini (2013), who focused the sample size at which the estimate does not leave the COS again. The definitions used in the current study does not preclude the estimate or interval bounds from leaving the COS again, but one can instead decide on a threshold for the probability that they do. Finally, since most previous simulation studies did not directly compare Bayesian and frequentist approaches (van de Schoot et al., 2017), the current study will include frequentist models that will serve as a point of reference for comparisons.

Due to the computational demands of running Monte Carlo simulations with Bayesian models, the different ρ will be limited to 0.1, 0.2, 0.3, and 0.4. A ρ of 0.2 is in line with previous estimates of the average effect size in psychology (Richard et al., 2003; Stanley et al., 2018), and the range considered is in line with newer guidelines for what constitutes small, medium, and large effect sizes (Gignac and Szodorai, 2016; Funder and Ozer, 2019). Furthermore, the maximum sample size will be constrained to 500, since the influence of Bayesian priors is expected to diminish as the sample size increases (van de Schoot et al., 2015; Stefan et al., 2020).

## 2. Materials and methods

### 2.1. Data generation procedure

The Monte Carlo simulation approach used in the current study largely mirrors that used by Schönbrodt and Perugini (2013), with two notable exceptions. First, the generation of bootstrap samples from the simulated population data proved to be a significant computational bottleneck. Second, due to the non-trivial computational demands of Markov chain Monte Carlo (MCMC) sampling for the Bayesian models, the number of bootstrap replications had to be limited to 10,000. To ameliorate these issues, all necessary data was generated and saved to disk prior to running the models, according to the following procedure:

1. Set the *outer* seed and generate one million rows of bivariate normal data x and y with a specified correlation ρ . This is the population data.
2. Set the *inner* seed and randomly select and remove an initial 10 samples from the population data. Save samples to disk.
3. Set the *inner* seed and randomly select an additional sample from the remaining population data. Add to the previous samples and save to disk.
4. Repeat step 3 until the sample size is 500.
5. Repeat steps 1–4 10,000 times. These are the bootstrap replications.
6. Repeat steps 1–5 for each of ρ = 0.1, 0.2, 0.3, and 0.4.

By setting the *outer* seed depending on ρ , the same population data will always be generated in each bootstrap replication, while setting the *inner* seed depending on both ρ and the current bootstrap replication ensures that different samples and thus a different trajectory is generated during each bootstrap replication. The population data was generated using the mvrnorm function from the R package MASS (version 7.3–57) running on R version 4.2.1. Each individual data file was saved in JavaScript Object Notation (JSON) format, as this is the preferred data format for CmdStan (see the following section). With 491 different sample sizes (*n* = 10 to *n* = 500), four different ρ , and 10,000 replications, the outlined procedure generated a total of 19.64 million JSON files, which took approximately 100 h on a 12 CPU core Linux workstation.

### 2.2. Bayesian models

A linear regression approach with a Gaussian likelihood was used to estimate bivariate correlations. A *Normal* (0, 2.5) prior was used for β and a *Cauchy* (0, 1) prior was used for σ . The estimated correlation was constrained to fall between −1 and 1 by putting a *Beta* ( α , β ) prior on the transformed parameter ( β + 1)/2 (Gelman et al., 2014, p. 317). The prior could then be made more or less informative by varying the values of α and β .

**Figure 1:**
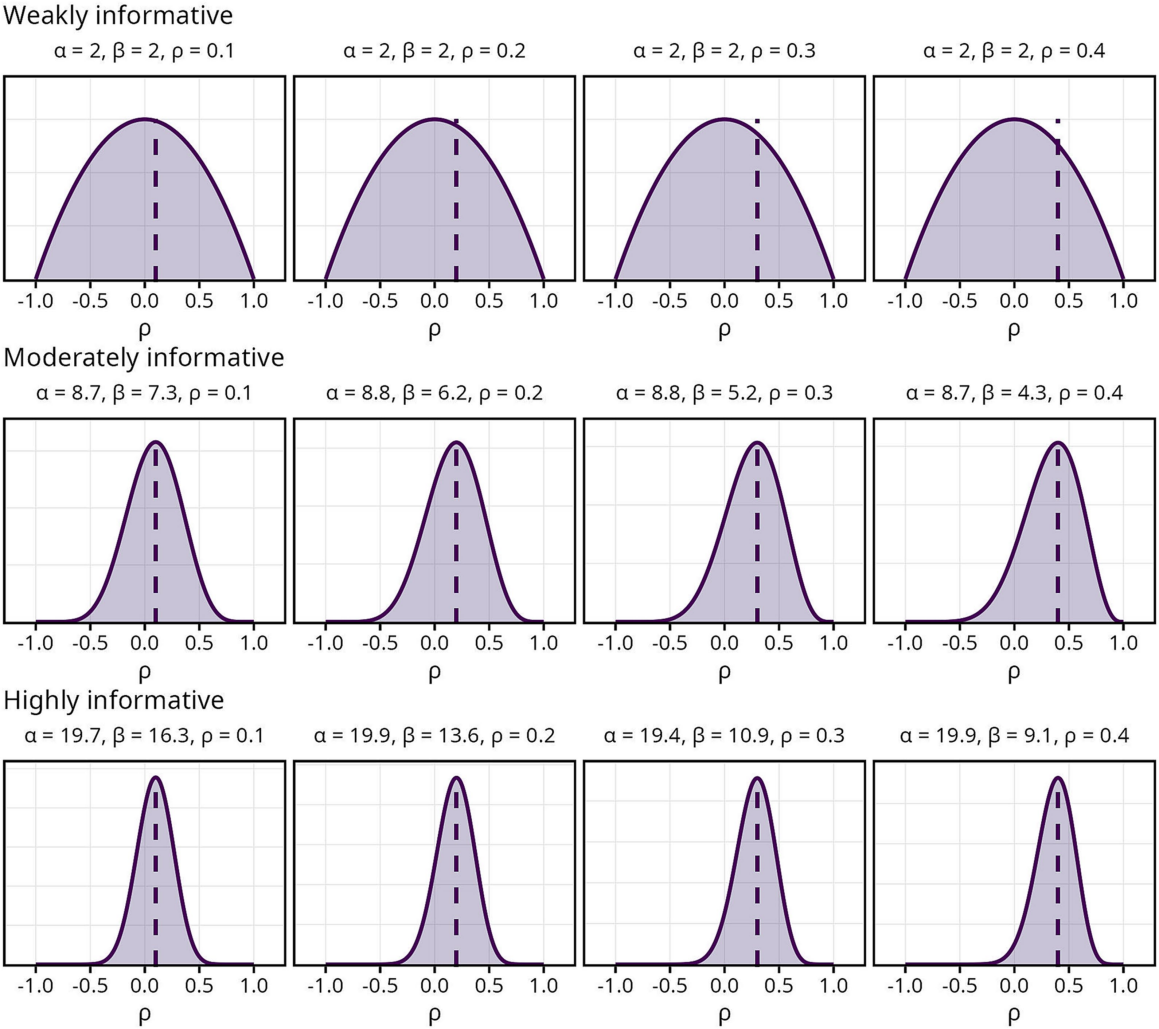
Overview of Bayesian priors. α and β indicate the parameters used for each prior, dashed lines indicate the population correlation coefficient ρ .

Three sets of priors, each reflecting three levels of informativeness, were used in the current study. The first, a *Beta* (2, 2) prior, was labeled “weakly informative.” Since it was centered around zero regardless of ρ , with equally diminishing probability mass on either side giving less credibility to extreme values, it should have a small regularizing effect on the estimate. The remaining two sets of priors, labeled “moderately informative” and “highly informative,” respectively, were constructed such that the mode of the distribution was centered around ρ . They differed in width and thus in how much credibility was assigned to values away from ρ , with the moderately informative prior having a wider distribution than the highly informative. The priors along with their respective α and β values are visualized in Figure 1.

It is important to note that “levels of informativeness” is used here in a similar fashion to “degree of prior knowledge.” Thus, the more informative priors outlined above reflect a state of more knowledge about the true distribution of ρ . A prior can also be informative in the sense of having a specific impact on the posterior *without* reflecting actual knowledge about the parameter. A narrow prior centered around −0.2, for instance, could be considered highly informative, but would not reflect prior knowledge.

All Bayesian models were specified using Stan (v2.31.0) and compiled into C++ executable programs using CmdStan (Lee et al., 2017). Sampling was carried out using four chains of 5,000 MCMC iterations each, after discarding 1,000 warm-up iterations. Step size was set to 0.05; all other settings remained at default values. Diagnostic information and posterior summaries — the posterior mean along with 66, 90 and 95% intervals based on percentiles — were obtained using CmdStan utility functions. A 95% interval was included due its close association with frequentist statistics, whereas the 90 and 66% intervals may be interpreted as being “very likely” and “likely,” respectively, to contain the true estimate (Mastrandrea et al., 2011).

### 2.3. Frequentist models

A custom C++ program, using the Armadillo library for linear algebra and scientific computing (Sanderson and Curtin, 2016) and JSON for Modern C++,^1^ was written to efficiently estimate the sample correlation coefficient. The program takes a JSON file as input and outputs the estimated sample correlation as well as 66, 90 and 95% intervals based on percentiles calculated using the Fisher *z*-transformation.

### 2.4. Monte Carlo simulation procedure

In total, 58.91 million Bayesian models and 19.64 million frequentist were estimated. All models were run as C++ executable programs via Linux shell scripts that supplied the JSON data files in parallel using GNU Parallel (Tange, 2011). Computations for the Bayesian models were carried out on a 32 CPU core node on the Tetralith high-performance computing (HPC) cluster located at the National Supercomputer Centre, Linköping University, Sweden.^2^ The entire computational environment required for running the simulations was packaged into a Singularity container,^3^ which is an open source, secure way to capture and distribute software and computational environments (Kurtzer et al., 2017). The Singularity container was built locally on a Linux workstation and uploaded to the Tetralith HPC cluster.

Simulations took approximately 75 h to run for each ρ and prior, with a total runtime of approximately 900 h (28 000 core hours), for the Bayesian models. All Gelman-Rubin convergence statistics ( $\hat{R}$ ) were < 1.00, indicating that all MCMC chains mixed well (Vehtari et al., 2021), and the average effective sample size (ESS) was 16,575, well above the recommended cutoff of 400 (Zitzmann and Hecht, 2019). Detailed MCMC diagnostic information is available in the Supplementary material. The total runtime for the frequentist models was negligible in comparison and was carried out locally on a 12 CPU core Linux workstation.

## 3. Results

**Figure 2:**
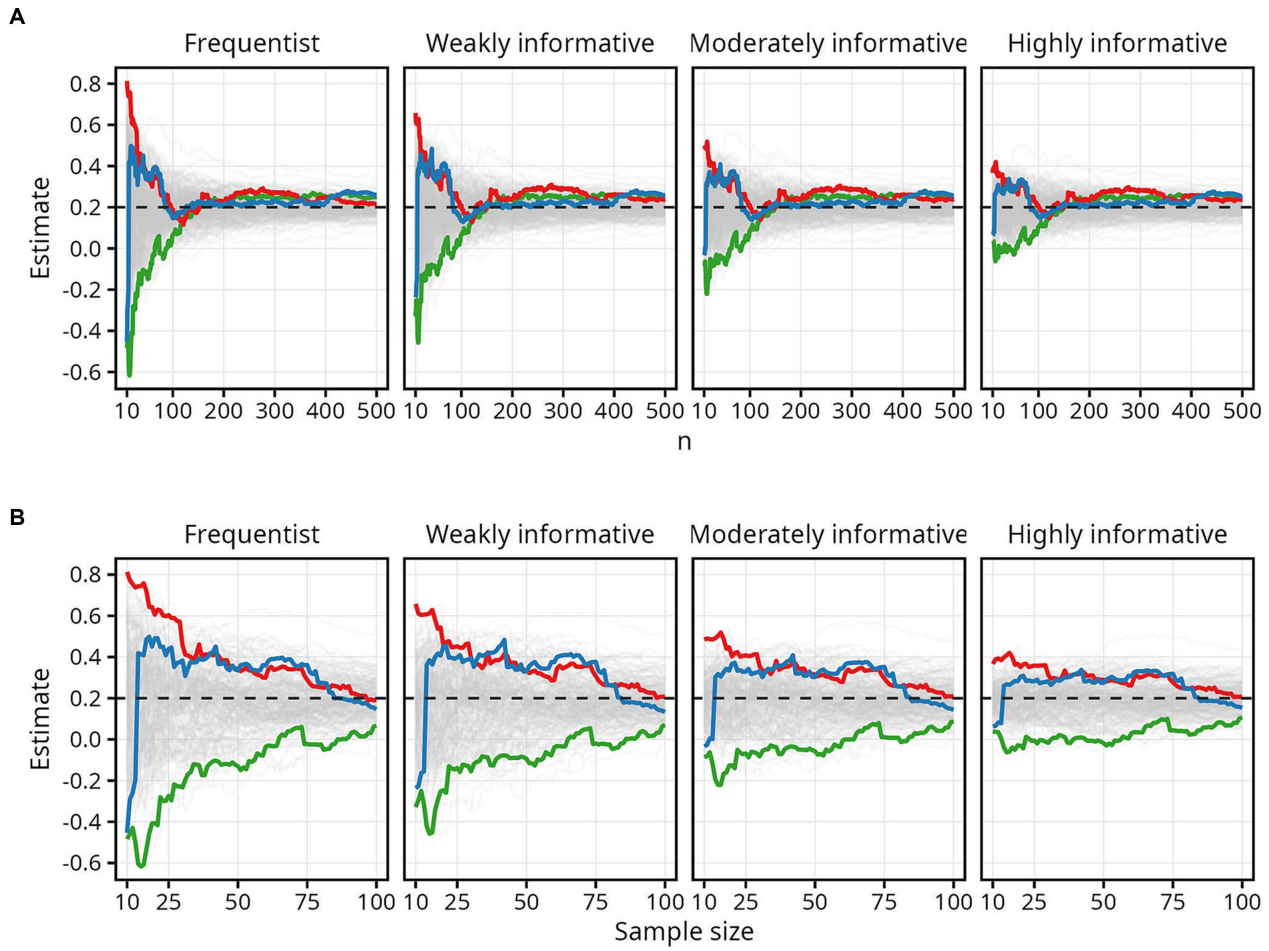
Examples of extreme trajectories. The colored lines highlight three simulated trajectories to illustrate the chaotic nature of estimates at small sample sizes. **(A)** Results for all sample sizes. **(B)** Results up until *N* = 100.

The chaotic nature of estimates at small sample sizes is illustrated in Figure 2, which traces every 50th simulated trajectory for ρ=0.2 for all four models. Three extreme trajectories are highlighted: the trajectory with the highest estimate (red line), lowest estimate (green line), and the trajectory with the largest difference between the highest and lowest estimate (blue line).

The red line begins with an estimate of about 0.8 at *N* = 10 — remarkably higher than the actual ρ of 0.2 — then tapers off toward ρ . The green line shows the opposite, with the estimate fluctuating between around −0.6 and − 0.4 at sample sizes up to 20, before slowly approaching ρ . The blue line shows how estimates can fluctuate rapidly and dramatically from negative to positive. Here, the estimate changes from about −0.4 to about 0.5 with just a small increase — from 10 to 20 — in sample size. While the overall pattern of the trajectories is the same for both the frequentist and the Bayesian models, Figure 2 illustrates the impact the more informative Bayesian priors have on restricting the range of possible estimates at sample sizes up to around 100.

### 3.1. Sample size required to obtain an estimate in the correct direction

**Figure 3:**
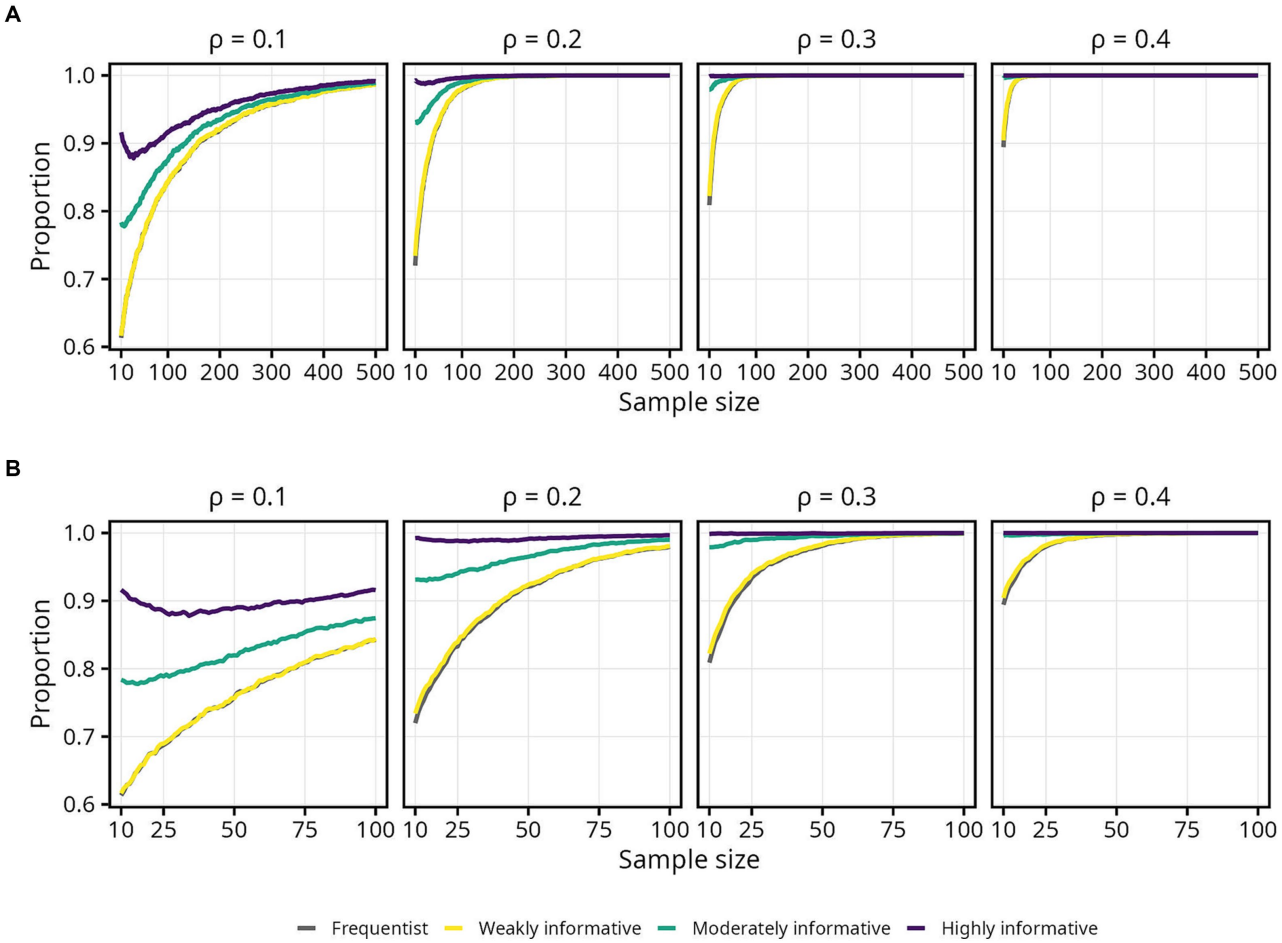
Proportion of sample estimates above zero for each population correlation coefficient ρ . Lines represent the aggregated proportion across 10,000 replications for each model. **(A)** Results for all sample sizes. **(B)** Results up until *N* = 100.

The proportion of estimates in the correct direction was, as expected, highly influenced by ρ , and the difference in required sample size when moving from ρ = 0.2 to ρ = 0.1 was pronounced. Interestingly, the proportion of estimates in the correct direction decreased slightly for highly informative model, as sample size increased from 10 to around 100. Differences between the frequentist and weakly informative models were negligible across all sample sizes and effect sizes (Figure 3).

Since obtaining an estimate in the correct direction is essential, most researchers will likely aim for a higher probability. Assuming a typical effect size of ρ = 0.2 and 95% probability of obtaining an estimate in the correct direction, the required sample size was 66 for the frequentist and weakly informative models, 35 (47% decrease) for the moderately informative model, and 10 or less (85% decrease or more) for the highly informative model. If instead assuming a smaller effect size of ρ = 0.1, all else equal, the required sample sizes were 269 for the frequentist and weakly informative models, 239 (11% decrease) for the moderately informative model, and 200 (26% decrease) for the highly informative model. An overview of sample sizes required for obtaining specific proportions of estimates in the correct direction, for each population ρ and model, is presented in Table 1.

**Table 1:** Sample size required for obtaining a specific proportion (*P*) of estimates in the correct direction, for different *ρ* and models.

ρ	*P*	Model			
		Frequentist	Weakly informative	Moderately informative	Highly informative
0.1	0.80	70	70^a^	35 (−50%)	< 10 (−86%)^b^
0.2	0.80	20	19 (−5%)	< 10 (−50%)^b^	< 10 (−50)^b^
0.3	0.80	10	10^a^	< 10^b^	< 10^b^
0.4	0.80	< 10^b^	< 10^b^	< 10^b^	< 10^b^
0.1	0.90	158	157 (−1%)	135 (−15%)	10 (−94%)
0.2	0.90	42	41 (−2%)	10 (−76%)	< 10 (−76%)^b^
0.3	0.90	19	18 (−5%)	< 10 (−47%)^b^	< 10 (−47%)^b^
0.4	0.90	11	10 (−9%)	< 10 (−9%)^b^	< 10 (−9%)^b^
0.1	0.95	269	268^a^	239 (−11%)	189 (−30%)
0.2	0.95	66	66^a^	35 (−47%)	< 10 (−85%)^b^
0.3	0.95	30	30^a^	< 10 (−67%)^b^	< 10 (−67%)^b^
0.4	0.95	17	16 (−6%)	< 10 (−41%)^b^	< 10 (−41%)^b^

Percentage difference from the frequentist model is presented within parenthesis. ^a^Less than 1% difference from frequentist model; no percentage change calculated. ^b^Required sample size less than 10; numbers represent upper bound.

### 3.2. Sample size required to obtain an estimate robustly different from zero

In contrast to differences in the proportion of estimates in the correct direction, differences in the proportion of estimates robustly different from zero were most pronounced at medium to large effect sizes. The differences between the frequentist and weakly informative models were small, although the regularizing effect of the weakly informative prior resulted in the weakly informative models always requiring a slightly larger sample size than the frequentist models to obtain the same proportion of estimates robustly different from zero. As expected, sample sizes for the frequentist model resembled those from a frequentist power analysis.^4^

Assuming a typical effect size of ρ = 0.2 and 80% probability of the estimate being robustly different from zero — akin to a statistical power of 80% in the frequentist approach — the required sample sizes were 190, 205 (8% increase), 186 (2% decrease), and 163 (14% decrease) for the frequentist, weakly, moderately, and highly informative models, respectively. If instead assuming ρ = 0.1, all models required sample sizes >500, and if assuming ρ = 0.3, the required sample sizes were 85, 90 (6% increase), 75 (12% decrease), and 53 (38% decrease) for the frequentist, weakly, moderately, and highly informative models, respectively. An overview of sample size required for obtaining different proportions of estimates robustly different from zero, using a 95% interval, is presented in Table 2. Details for 90 and 66% intervals are presented in Supplementary Figures S1, S2 as well as Supplementary Tables S3, S4.

**Table 2:** Sample size required for obtaining a specific proportion (*P*) of estimates robustly different from zero, using a 95% interval, for different *ρ* and models.

*ρ*	*P*	Model			
		Frequentist	Weakly informative	Moderately informative	Highly informative
0.1	0.80	> 500	> 500^a^	> 500^a^	> 500^a^
0.2	0.80	190	205 (+8%)	186 (−2%)	163 (−14%)
0.3	0.80	85	90 (+6%)	75 (−12%)	53 (−38%)
0.4	0.80	46	50 (+9%)	34 (−26%)	12 (−74%)
0.1	0.90	> 500	> 500^a^	> 500^a^	> 500^a^
0.2	0.90	256	272 (+6%)	253 (−1%)	228 (−11%)
0.3	0.90	113	119 (+5%)	103 (−9%)	78 (−31%)
0.4	0.90	60	64 (+7%)	49 (−18%)	24 (−60%)
0.1	0.95	> 500	> 500^a^	> 500^a^	> 500^a^
0.2	0.95	313	332 (+6%)	309 (−1%)	285 (−9%)
0.3	0.95	136	144 (+6%)	127 (−7%)	104 (−24%)
0.4	0.95	73	78 (+7%)	63 (−14%)	37 (−49%)

Percentage difference from the frequentist model is presented within parenthesis. Robustly different from zero is defined here as the lower bound of the associated interval being above zero. ^a^Required sample size above 500 for all models; no percentage change calculated.

### 3.3. Sample size required to obtain an estimate within an acceptable range

#### 3.3.1. Proportion of estimates within the COS

**Figure 4:**
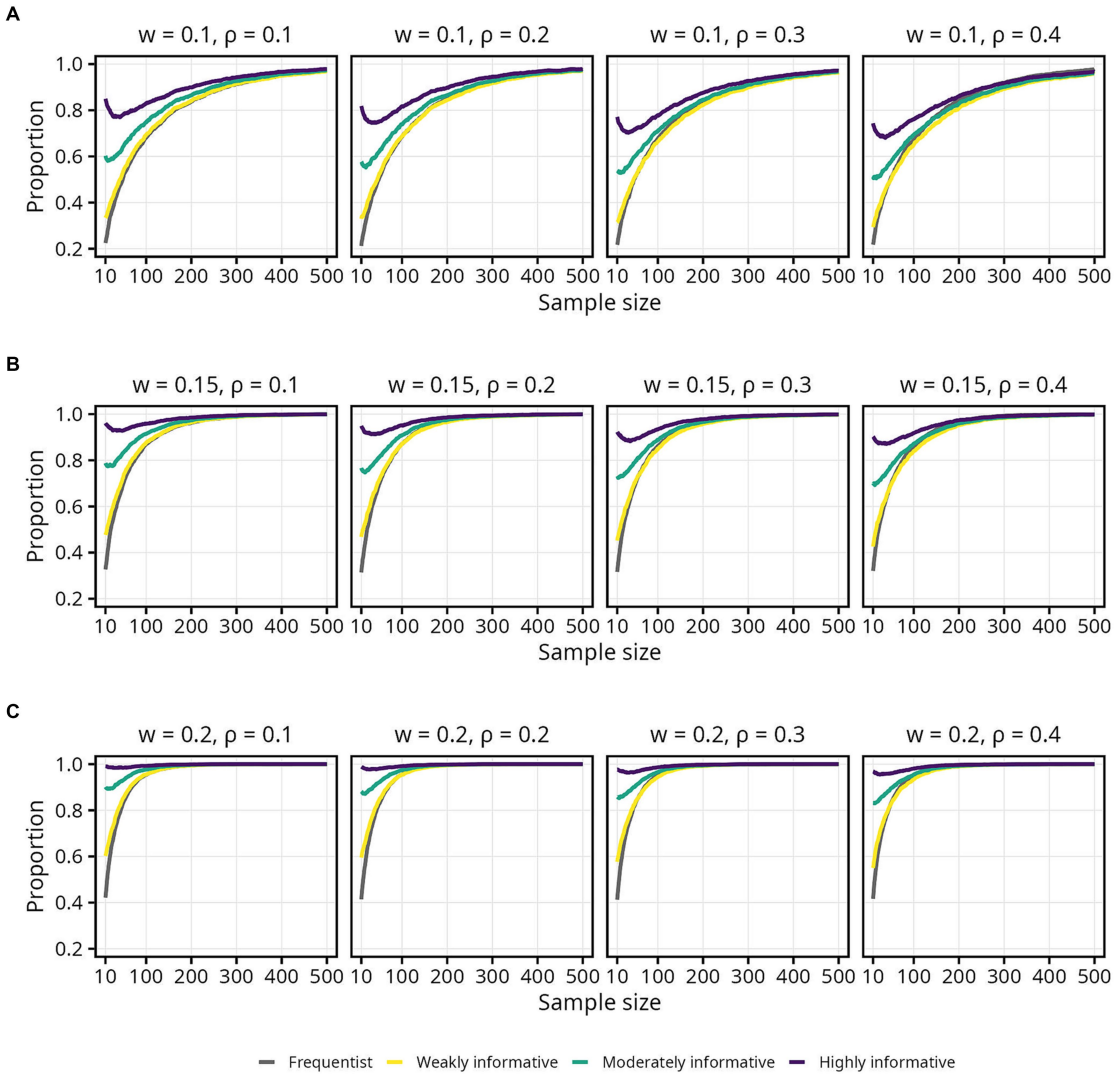
Proportion of sample estimates within different widths (*w*) of the corridor of stability (COS) for each population correlation coefficient ρ . Lines represent the aggregated proportion across 10,000 replications for each model. **(A)** *w* = 0.1. **(B)** *w* = 0.15. **(C)** *w* = 0.2.

**Figure 5:**
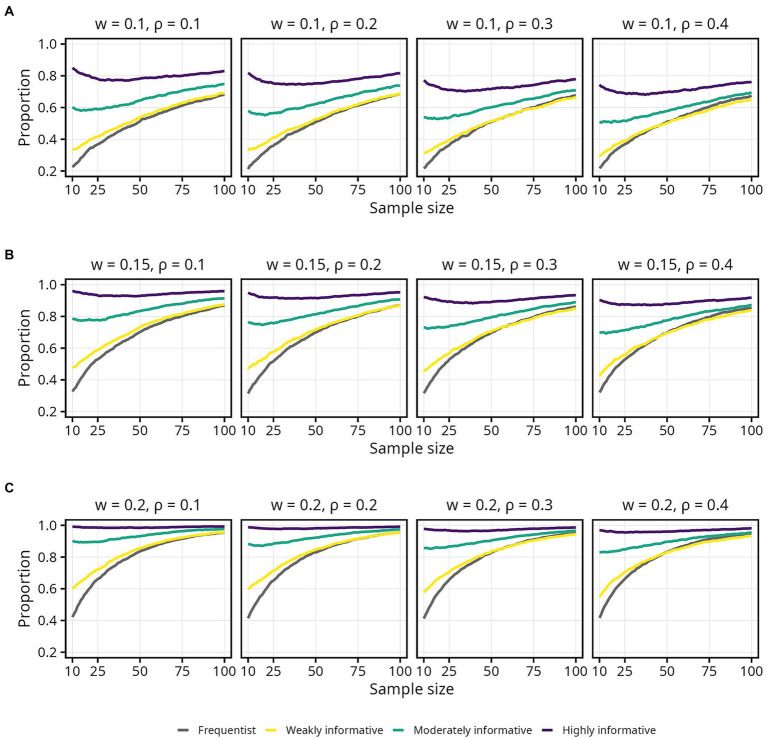
Proportion of sample estimates within different widths (*w*) of the corridor of stability (COS) for each population correlation coefficient ρ . Lines represent the aggregated proportion across 10,000 replications for each model. Only showing sample sizes up to *N* = 100. **(A)** *w* = 0.1. **(B)** *w* = 0.15. **(C)** *w* = 0.2.

The impact of informative Bayesian priors on the proportion of estimates inside the COS was relatively pronounced at smaller sample sizes, but quickly tapered off as sample size increased (Figures 4, 5). The regularizing effect of a weakly informative prior seems to result in weakly informative models having a slightly higher proportion of estimates inside the COS, compared to the frequentist models, but again mainly for sample sizes up until around 50 (Figure 5). For the widest COS (*w* = 0.2), the proportion of estimates inside the COS never dropped below 0.8 for either the moderately or highly informative models. Furthermore, a slight tendency toward a *lower* proportion of estimates inside the COS with increasing ρ was observed for the Bayesian models (Figure 5A). Note also how, again, the proportion of estimates inside the COS decreased slightly between sample sizes 10 to 100 for the moderately and highly informative models (Figure 5A).

Assuming a typical effect size of ρ=0.2 and accepting only small fluctuations (*w* = 0.1) while aiming for 80% probability of the estimate being inside the COS, the required sample sizes were 158, 159 (1% increase), 139 (12% decrease), and 10 (94% decrease) for the frequentist, weakly, moderately, and highly informative models, respectively. If one wants to be more certain — 95% probability that the estimate falls inside the COS — while still tolerating only small fluctuations (*w* = 0.1), the required sample sizes were 363, 383 (6% increase), 364 (less than 1% increase), and 319 (12% decrease) for the frequentist, weakly, moderately, and highly informative models, respectively. An overview of the sample size required for different ρ , proportions, and COS widths is presented in Table 3.

**Table 3:** Sample size required for obtaining a specific proportion (*P*) of estimates within a specific width (*w*) of the corridor of stability, for different *ρ* and models.

*ρ*	*P*	*w*	Model			
			Frequentist	Weakly informative	Moderately informative	Highly informative
0.1	0.80	0.20	44	38 (−14%)	< 10 (−77%)^b^	< 10 (−77%)^b^
0.2	0.80	0.20	44	39 (−11%)	< 10 (−77%)^b^	< 10 (−77%)^b^
0.3	0.80	0.20	45	42 (−7%)	< 10 (−78%)^b^	< 10 (−78%)^b^
0.4	0.80	0.20	45	44 (−2%)	10 (−78%)	< 10 (−78%)^b^
0.1	0.80	0.15	75	69 (−8%)	35 (−53%)	< 10 (−87%)^b^
0.2	0.80	0.15	75	73 (−3%)	44 (−41%)	< 10 (−87%)^b^
0.3	0.80	0.15	76	78 (+3%)	54 (−29%)	< 10 (−87%)^b^
0.4	0.80	0.15	76	83 (+9%)	61 (−20%)	< 10 (−87%)^b^
0.1	0.80	0.10	163	158 (−3%)	137 (−16%)	10 (−94%)
0.2	0.80	0.10	158	159 (+1%)	139 (−12%)	10 (−94%)
0.3	0.80	0.10	169	182 (+8%)	158 (−7%)	118 (−30%)
0.4	0.80	0.10	166	193 (+16%)	174 (+5%)	134 (−19%)
0.1	0.90	0.20	69	65 (−6%)	10 (−86%)	< 10 (−86%)^b^
0.2	0.90	0.20	70	69 (−1%)	36 (−49%)	< 10 (−86%)^b^
0.3	0.90	0.20	71	73 (+3%)	46 (−35%)	< 10 (−86%)^b^
0.4	0.90	0.20	70	77 (+10%)	54 (−23%)	< 10 (−86%)^b^
0.1	0.90	0.15	123	119 (−3%)	85 (−31%)	< 10 (−92%)^b^
0.2	0.90	0.15	118	118^a^	94 (−20%)	10 (−92%)
0.3	0.90	0.15	124	130 (+5%)	108 (−13%)	10 (−92%)
0.4	0.90	0.15	123	141 (+15%)	123^a^	10 (−92%)
0.1	0.90	0.10	276	273 (−1%)	246 (−11%)	203 (−26%)
0.2	0.90	0.10	260	266 (+2%)	244 (−6%)	204 (−22%)
0.3	0.90	0.10	284	301 (+6%)	284^a^	242 (−15%)
0.4	0.90	0.10	267	314 (+18%)	295 (+10%)	263 (−1%)
0.1	0.95	0.20	96	94 (−2%)	64 (−33%)	< 10 (−90%)^b^
0.2	0.95	0.20	95	97 (+2%)	71 (−25%)	< 10 (−89%)^b^
0.3	0.95	0.20	99	106 (+7%)	83 (−16%)	< 10 (−90%)^b^
0.4	0.95	0.20	100	114 (+14%)	97 (−3%)	< 10 (−90%)^b^
0.1	0.95	0.15	173	169 (−2%)	146 (−16%)	< 10 (−94%)^b^
0.2	0.95	0.15	162	168 (+4%)	142 (−12%)	96 (−41%)
0.3	0.95	0.15	174	185 (+6%)	162 (−7%)	121 (−30%)
0.4	0.95	0.15	174	196 (+13%)	184 (+6%)	146 (−16%)
0.1	0.95	0.10	396	400 (+1%)	377 (−5%)	326 (−18%)
0.2	0.95	0.10	363	383 (+6%)	364^a^	319 (−12%)
0.3	0.95	0.10	393	436 (+11%)	416 (+6%)	380 (−3%)
0.4	0.95	0.10	370	467 (+26%)	444 (+20%)	399 (+8%)

Percentage difference from the frequentist model is presented within parenthesis. ^a^Less than 1% difference from frequentist model; no percentage change calculated. ^b^Required sample size less than 10; numbers represent upper bound.

#### 3.3.2. Proportion of intervals within the COS

**Figure 6:**
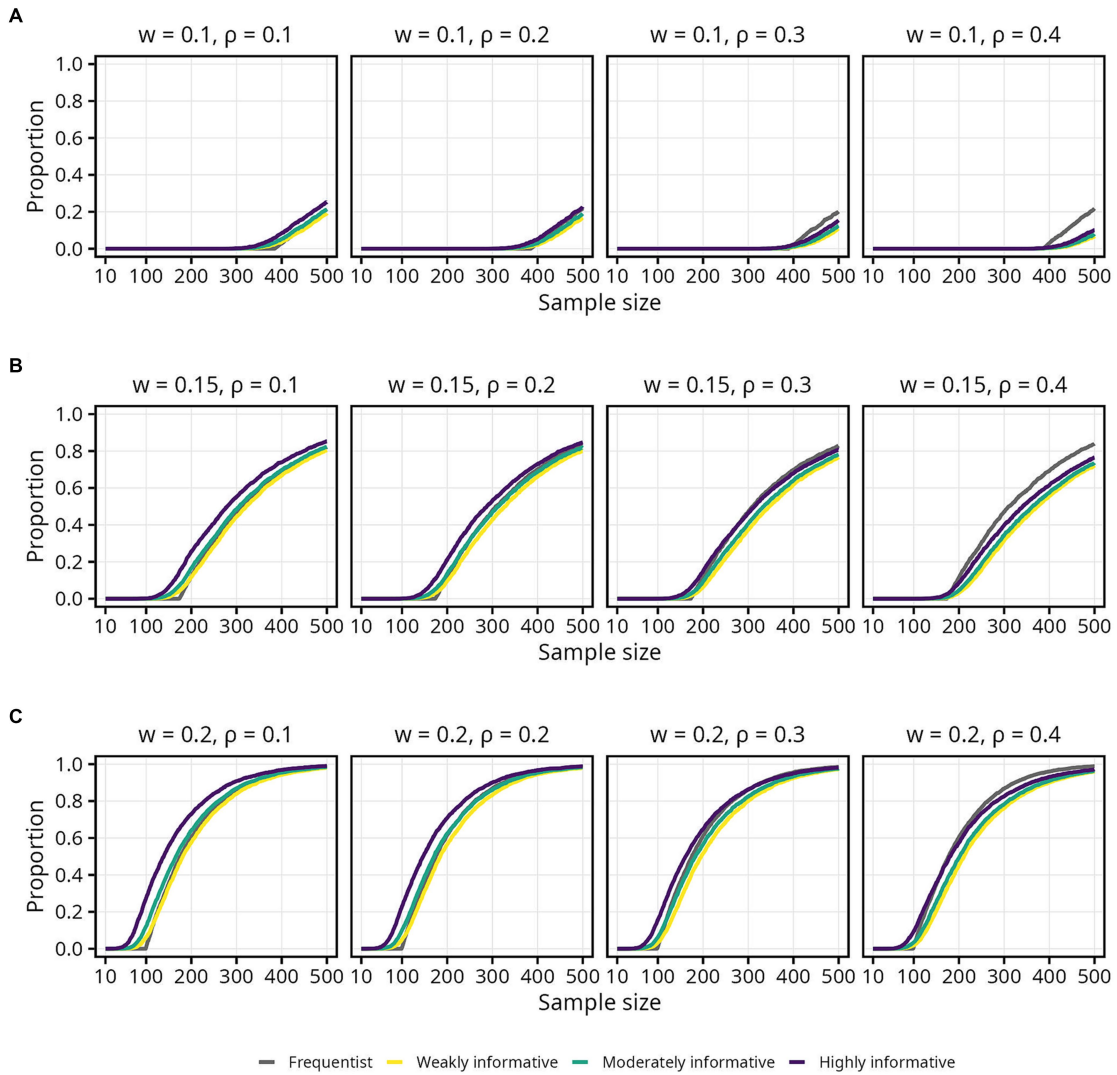
Proportion of 95% intervals within different widths (*w*) of the corridor of stability (COS) for each population correlation coefficient ρ . Lines represent the aggregated proportion across 10,000 replications for each model. **(A)** *w* = 0.1. **(B)** *w* = 0.15. **(C)** *w* = 0.2.

**Figure 7:**
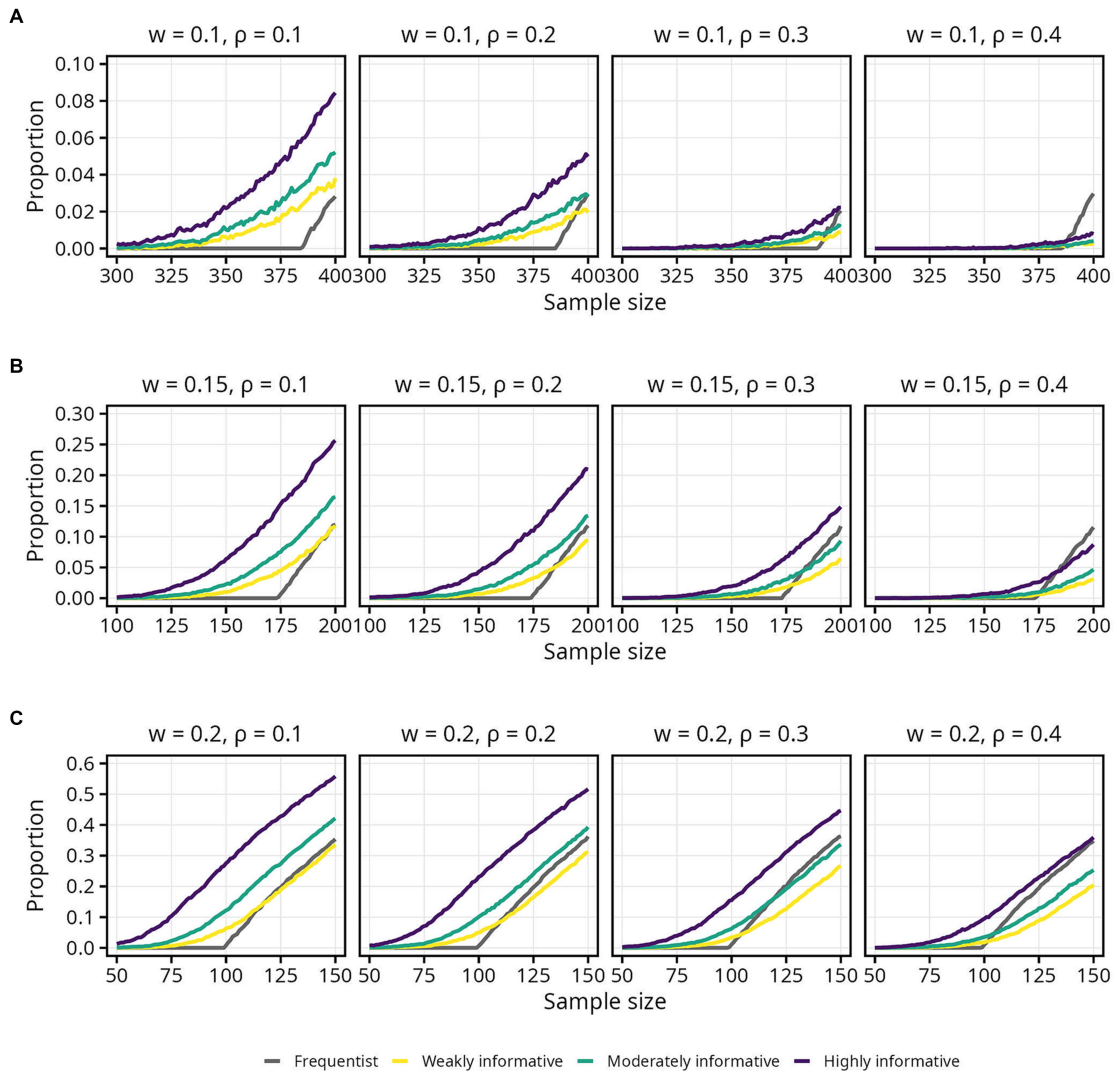
Proportion of 95% intervals within different widths (*w*) of the corridor of stability (COS) for each population correlation coefficient ρ . Lines represent the aggregated proportion across 10,000 replications for each model. Only showing specific sample sizes. **(A)** *w* = 0.1. **(B)** *w* = 0.15. **(C)** *w* = 0.2.

There was a gradual, S-shaped increase in the proportion of intervals within the COS for the Bayesian models, and a sharp and linear increase for the frequentist models (Figure 6). Zooming in, the sharp increase for the frequentist models appears to always begin at the same sample size for any given *w*, regardless of ρ . The proportion of intervals within the COS is, on the other hand, higher at smaller ρ for the Bayesian models, at any given sample size (Figure 7). The proportion of intervals within the COS is becomes highest for the frequentist models when ρ = 0.4 as sample size approaches 500 (Figure 7, final panel of each row).

Overall, the weakly informative model always required a higher sample size than the frequentist model, and reductions in required sample size was primarily seen for the highly informative models when ρ = 0.1 and 0.2. In several cases the required sample size was above 500. For instance, if accepting only small fluctuations (*w* = 0.1) while aiming for 80% probability of the 95% interval being inside the COS, the required sample was above 500 for all models, regardless of ρ . Widening the COS to *w* = 0.15 still required a sample size between 450 and 500, with the weakly informative model always requiring a larger (3–7%) sample than the frequentist model. For the widest COS width, *w* = 0.20, assuming a typical effect size of ρ=0.2 and still aiming for 80% probability of the 95% interval being inside the COS, the required sample sizes were 262, 281 (7% increase), 263 (less than 1% increase), and 238 (9% decrease) for the frequentist, weakly, moderately, and highly informative models, respectively. An overview of the sample size required for different ρ , proportions, and COS widths is presented in Table 4. Details for 90 and 66% intervals are presented in Supplementary Figures S3, S4 as well as Supplementary Tables S5, S6.

**Table 4:** Sample size required for obtaining a specific proportion (*P*) of 95% intervals within a specific width (*w*) of the corridor of stability, for different *ρ* and models.

*ρ*	*P*	*w*	Model			
			Frequentist	Weakly informative	Moderately informative	Highly informative
0.1	0.80	0.20	271	280 (+3%)	261 (−4%)	230 (−15%)
0.2	0.80	0.20	262	281 (+7%)	263^a^	238 (−9%)
0.3	0.80	0.20	265	301 (+14%)	287 (+8%)	260 (−2%)
0.4	0.80	0.20	264	320 (+21%)	309 (+17%)	284 (8%)
0.1	0.80	0.15	482	496 (+3%)	476 (−1%)	449 (−7%)
0.2	0.80	0.15	472	498 (+6%)	482 (+2%)	451 (−4%)
0.3	0.80	0.15	474	> 500 (+5%)^c^	> 500 (+5%)^c^	492 (4%)
0.4	0.80	0.15	467	> 500 (+7%)^c^	> 500 (+7%)^c^	> 500 (7%)^c^
0.1	0.80	0.10	> 500	> 500^b^	> 500^b^	> 500^b^
0.2	0.80	0.10	> 500	> 500^b^	> 500^b^	> 500^b^
0.3	0.80	0.10	> 500	> 500^b^	> 500^b^	> 500^b^
0.4	0.80	0.10	> 500	> 500^b^	> 500^b^	> 500^b^
0.1	0.90	0.20	333	347 (+4%)	326 (−2%)	292 (−12%)
0.2	0.90	0.20	325	346 (+6%)	332 (+2%)	300 (−8%)
0.3	0.90	0.20	330	371 (+12%)	357 (+8%)	332 (1%)
0.4	0.90	0.20	324	400 (+23%)	387 (+19%)	360 (11%)
0.1	0.90	0.15	> 500	> 500^b^	> 500^b^	> 500^b^
0.2	0.90	0.15	> 500	> 500^b^	> 500^b^	> 500^b^
0.3	0.90	0.15	> 500	> 500^b^	> 500^b^	> 500^b^
0.4	0.90	0.15	> 500	> 500^b^	> 500^b^	> 500^b^
0.1	0.90	0.10	> 500	> 500^b^	> 500^b^	> 500^b^
0.2	0.90	0.10	> 500	> 500^b^	> 500^b^	> 500^b^
0.3	0.90	0.10	> 500	> 500^b^	> 500^b^	> 500^b^
0.4	0.90	0.10	> 500	> 500^b^	> 500^b^	> 500^b^
0.1	0.95	0.20	393	412 (+5%)	391 (−1%)	359 (−9%)
0.2	0.95	0.20	380	409 (+8%)	395 (+4%)	365 (−4%)
0.3	0.95	0.20	390	443 (+14%)	428 (+10%)	400 (+3%)
0.4	0.95	0.20	379	477 (+26%)	466 (+23%)	438 (+16%)
0.1	0.95	0.15	> 500	> 500^b^	> 500^b^	> 500^b^
0.2	0.95	0.15	> 500	> 500^b^	> 500^b^	> 500^b^
0.3	0.95	0.15	> 500	> 500^b^	> 500^b^	> 500^b^
0.4	0.95	0.15	> 500	> 500^b^	> 500^b^	> 500^b^
0.1	0.95	0.10	> 500	> 500^b^	> 500^b^	> 500^b^
0.2	0.95	0.10	> 500	> 500^b^	> 500^b^	> 500^b^
0.3	0.95	0.10	> 500	> 500^b^	> 500^b^	> 500^b^
0.4	0.95	0.10	> 500	> 500^b^	> 500^b^	> 500^b^

Percentage difference from the frequentist model is presented within parenthesis. *^a^*Less than 1% difference from frequentist model; no percentage change calculated. ^b^Required sample size above 500 for all models; no percentage change calculated. ^c^Required sample size above 500; numbers represent lower bound.

## 4. Discussion

The current study used Monte Carlo simulations to examine the impact of three different Bayesian priors, with varying degrees of informativeness, on the sample size required to conclude that an estimate is (1) in the correct direction, (2) robustly different from zero, and (3) within an acceptable range. The results showed that while Bayesian priors can have an appreciable impact, the impact differs for each of the three aims and depends to a large degree on ρ as well as on one’s threshold for probability and precision. Overall, and in line with expectations (van de Schoot et al., 2015; e.g., Stefan et al., 2020), the stabilizing effect of informative Bayesian priors was primarily observed at small sample sizes. Previous work has documented a robust negative correlation between sample size and effect size in psychological research, indicating that studies using small sample sizes tend to report exaggerated effects (Kühberger et al., 2014). The results from the current study, together with previous simulation work (Schönbrodt and Perugini, 2013), lends further credence to this observation; small sample size studies are indeed sailing in a “sea of chaos” (Lakens and Evers, 2014). As illustrated by Figure 2, the risk of committing a Type S error — reporting an estimate in the wrong direction — or a Type M error — reporting an exaggerated estimate — remains high until sample sizes approach 100. As Figure 2 further illustrates, however, this instability at small sample sizes can be constrained by informative Bayesian priors.

### 4.1. Weakly informative priors

The impact of an informative Bayesian prior obviously depends on its degree of informativeness. So-called weakly informative priors do not include any domain-specific information and are typically designed to have a small regularizing effect on the estimate. This regularizing effect is similar maximum likelihood approaches to regularization or penalization, which makes them desirable as a kind of “default” prior (Cole et al., 2014; Gelman et al., 2017). Using a weakly informative prior, thus, can be seen a way to “let the data speak” while also ruling out impossible or implausible values, which can take over the posterior distribution when the sample size is small (Gelman, 2009, p. 176).

Using a weakly informative prior had no impact on obtaining an estimate in the correct direction, and thus no impact on the risk of committing a Type S error, compared to a frequentist model. In terms of obtaining an estimate robustly different from zero, however, the regularizing effect of the weakly informative prior came into play. The weakly informative models always required a slightly larger sample size the frequentist models to obtain the same proportion of estimates robustly different from zero. At the same time, as shown in Figure 5, the weakly informative models had a higher proportion of estimates inside the COS compared to the frequentist models, thus decreasing the risk of committing a Type M error, at sample sizes up to around 50. As sample size increased this effect diminished, however, with the weakly informative models instead requiring a slightly larger sample size to obtain same proportion of estimates within COS as the frequentist models. For a specific and relatively small sample size range, the weakly informative models also had a higher proportion of intervals inside the COS compared to the frequentist models (Figure 7).

Taken together, a weakly informative prior seems particularly useful when conducting research with small sample sizes, given its ability to both stabilize and regularize parameter estimates. With larger sample sizes the stabilizing effect diminishes, but the regularizing effect may still be desirable.

### 4.2. Moderately and highly informative priors

**Figure 8:**
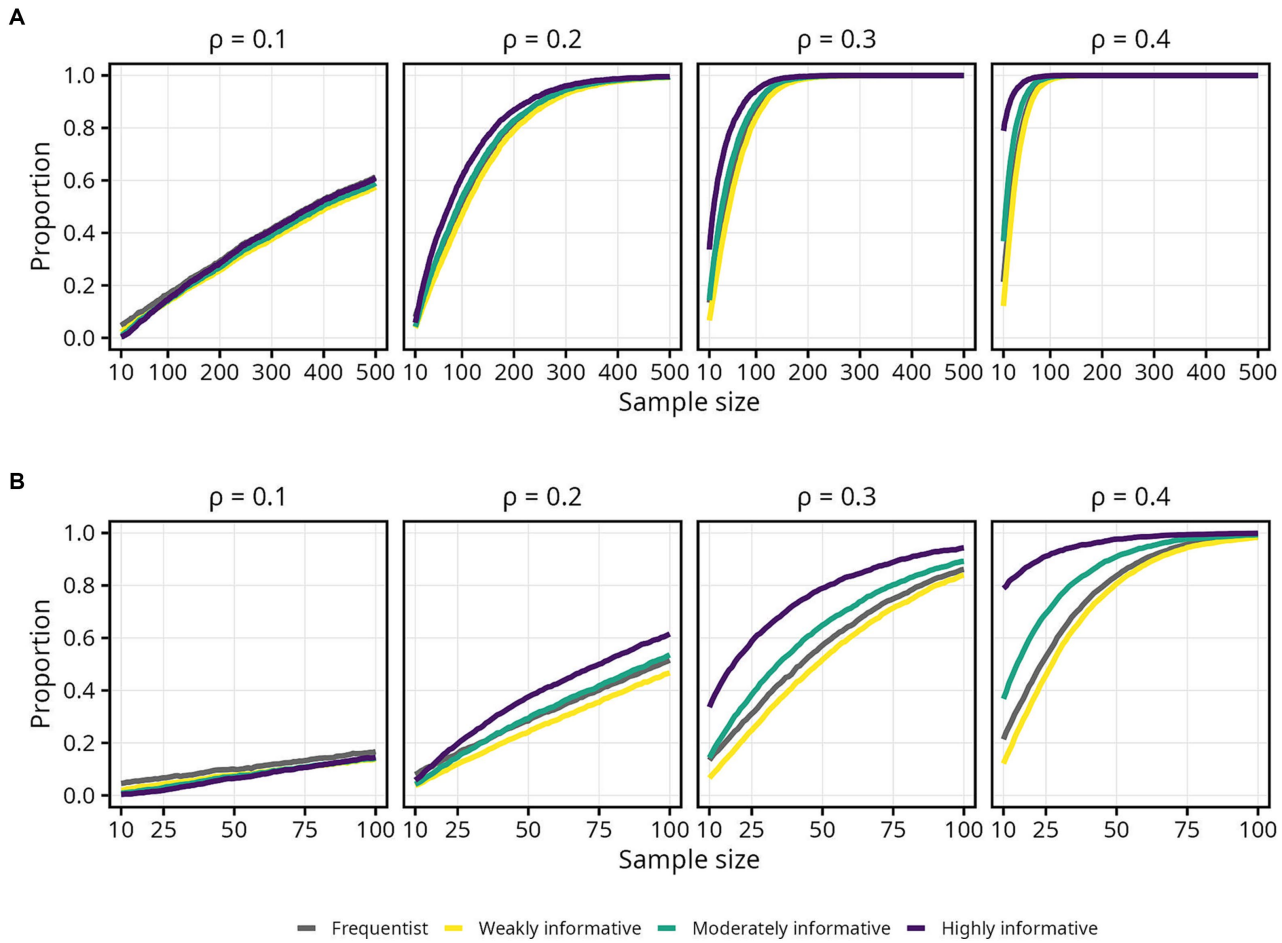
Proportion of lower 95% interval bound above zero for each population correlation coefficient ρ . Lines represent the aggregated proportion across 10,000 replications for each model. **(A)** Results for all sample sizes. **(B)** Results up until *N* = 100.

The impact of the moderately and highly informative priors was particularly evident in terms of obtaining an estimate in the correct direction, and thus decreasing the risk of a Type S error. Compared to the frequentist models, the moderately and in particular the highly informative models could reach the same proportion of estimates in the correct direction using considerably smaller samples. In terms of obtaining an estimate robustly above zero, these more informative priors had less impact. Still, an interesting observation was that the effect of a moderately and especially a highly informative prior on the proportion of estimates robustly above zero was more pronounced for larger ρ . In fact, at ρ = 0.1 and sample sizes up to around 100, the proportion of estimates robustly above zero was slightly *smaller* for the moderately and highly informative models compared to the frequentist model (Figure 8B). The reason for this is unclear and should be investigated in further detail. The impact of the moderately and highly informative priors on the proportion of estimates and intervals within an acceptable range depended both on ρ and on the COS width; a larger ρ led to *less* impact, and a wider COS led to *higher* impact.

Taken together, a moderately or highly informative prior can lower the sample size required to obtain a precise estimate, or conversely decrease the risk of committing a Type M error at a given sample size, but primarily for larger effect sizes and with less precision. Some caution is warranted, however, since careful reading of Tables 3, 4 reveals some unexpected findings. For instance, in Table 3, when *w* = 0.1, the proportion *p* = 0.95, and looking only at the frequentist model, the required sample sizes are 396, 363, 393, and 370 for ρ = 0.1, 0.2, 0.3, and 0.4, respectively. It is unclear why the required sample size should drop, then increase, then drop again, for increasing values of ρ . Since the same pattern was observed for both frequentist and Bayesian models, issues stemming from MCMC sampling can be ruled out.

One possible explanation is that the number of replications was too low to obtain stable results. Indeed, the jagged lines seen most prominently in Figures 3, 7 suggest that there is still appreciable variability in the aggregated estimates. Thus, additional replications may have been necessary to obtain more accurate simulation results. Previous work by Schönbrodt and Perugini (2013) and Lakens and Caldwell (2021) used 100,000 replications, and both offer convincing explanations for why such a high number is important. Although previous Bayesian simulation studies have used 5,000 replications (Brysbaert, 2019), 1,000 replications (Hox et al., 2012; van de Schoot et al., 2015), or even less (Holtmann et al., 2016), the 10,000 replications used in the current study may simply not be adequate. Unfortunately, 100,000 replications is not feasible within a reasonable time frame when estimating Bayesian models using MCMC unless substantial computational resources are available.

### 4.3. Limitations and future directions

Throughout the results section, ρ = 0.2 has served as a reference to the “typical effect size” in psychological research. Although based on a substantial amount of previous research (Richard et al., 2003; Stanley et al., 2018), it is nevertheless likely that this number is overestimated due to publication bias and the favoring of large and statistically significant effects (Funder and Ozer, 2019). Increased attention has been given to the dangers of a culture that demands large effects, and that accepting small effects as the norm is critical for reliable and reproducible psychological research (Götz et al., 2021). Thus, it seems reasonable to suspect that the typical effect size in psychological research lies somewhere between ρ = 0.1 and ρ = 0.2. The difference in required sample size when moving from ρ = 0.2 to ρ = 0.1 was quite drastic, but unfortunately no intermediate effect sizes were included, and thus no intermediate sample size requirements are available in the current study. Although the sample size requirements for obtaining an estimate either in the correct direction or robustly different from zero seem to be captured quite well using a nonlinear least squares model with an exponential link function, future work should keep this limitation in mind.

It should be reiterated that the priors used in this study were constructed and defined as weakly, moderately, and highly informative solely by the author. Recent work by Sarma and Kay (2020) shows that prior elicitation is influenced by both available information but also by statistical ideology and past experience. Interestingly, the authors found that while weakly informative priors are popular they are implemented inconsistently, and different researchers have their on view on what “weakly informative” should entail. They also found that researchers find it particularly difficult to elicit priors for complicated parameters, such as transformed coefficients. This is further complicated by the fact that in more complex models, there are several ways informative priors can be incorporated in order to increase stability and parameter accuracy (e.g., Zitzmann et al., 2021a,b). Taken together, detailed and transparent reasoning is key whenever Bayesian priors are used, even if they are “just” weakly informative. Future work should explore the impact of priors tailor-made for specific research questions and in specific contexts, utilizing both prior research findings, such as from meta-analyses and elicited through expert judgment, along with appropriate sensitivity analyses (Lakens and Evers, 2014; van de Schoot et al., 2017; Stefan et al., 2020). In addition, another avenue worth exploring is the impact of “incorrect” informative priors. In the context of the current study, an “incorrect” informative prior could be a prior with mode 0 and extremely narrow width. While such a prior is not informative in the sense of reflecting knowledge about the true parameter distribution, it still likely has a strong influence on the posterior distribution.

The kind of standardized, multivariate normal data used in the current study is likely not an accurate reflection of the variability present in real psychological data (Smid et al., 2020). Caution is therefore recommended when interpreting the results of the current study, and future work may benefit from further investigating the impact of Bayesian priors on estimates obtained from non-normal data as well as data with outliers (e.g., de Winter et al., 2016). Similarly, although not a focus of the current study, the Bayesian approach also allows for specifying different likelihood functions. Importantly, it has been argued that the prior can only fully be understood in the context of the likelihood, at least when using default priors such as weakly informative ones (Gelman et al., 2017). A Student’s T likelihood (Lange et al., 1989), for instance, may be particularly suitable for achieving more robust estimates when outliers are present.

The focus of the current work has been the bivariate correlation. Although a simple model, it forms the foundation of several more advanced statistical techniques, including factor analysis, structural equation models, and multiple regression (Rodgers and Nicewander, 1988; Goodwin and Leech, 2006). This ubiquity has led the bivariate correlation to be described as a cornerstone of statistical analysis (Olkin and Finn, 1995). Nevertheless, future work should examine the impact of Bayesian priors on more sophisticated models. Finally, future work may also want to consider other approaches to defining an acceptable range, such as using the region of practical equivalence (Kruschke, 2018).

### 4.4. Conclusion

The current study found that bivariate correlation estimates were highly unstable, and consequently that the impact of informative Bayesian priors was most evident, at sample sizes up to around 100. Owing to its combined stabilizing and regularizing effect, a weakly informative prior is particularly useful when conducting research with small samples. For larger samples, and despite the slight *increase* in required sample size compared to frequentist models, its regularizing effect may still prove valuable enough to warrant its use. Whether more informative Bayesian priors can relax sample size requirements compared to a frequentist model is highly dependent on one’s goal, be it obtaining an estimate merely in the correct direction or a high precision estimate whose associated interval falls within a narrow range, and threshold for probability. Still, in settings where small samples are expected, such as when participant recruitment is expensive or otherwise difficult, using informative Bayesian priors can help ensure that obtained estimates are in line with realistic, real-world expectations rather than succumbing to chaos.

## Data availability statement

The original contributions presented in the study are included in the article/Supplementary material, further inquiries can be directed to the corresponding author.

## Author contributions

CD: Conceptualization, Formal analysis, Investigation, Methodology, Project administration, Software, Visualization, Writing – original draft, Writing – review & editing.

## Funding

This study was partially funded by the Regional Forensic Psychiatric Clinic in Växjö, Sweden. All Bayesian computations were carried out using resources available at the National Supercomputer Centre in Linköping, Sweden, through the National Academic Infrastructure for Supercomputing in Sweden (NAISS), grant agreement no. 2022/22-1182. NAISS is partially funded by the Swedish Research Council through grant agreement no. 2018-05973.

## Conflict of interest

The author declares that the research was conducted in the absence of any commercial or financial relationships that could be construed as a potential conflict of interest.

## Publisher’s note

All claims expressed in this article are solely those of the authors and do not necessarily represent those of their affiliated organizations, or those of the publisher, the editors and the reviewers. Any product that may be evaluated in this article, or claim that may be made by its manufacturer, is not guaranteed or endorsed by the publisher.

## Footnotes

^1^https://json.nlohmann.me/

^2^https://www.nsc.liu.se/

^3^https://singularity.hpcng.org

^4^Using, for instance, inverse tangent approximation: $N=1+\rho /\left(2\times \left(\beta -atan\left(\rho \right)\right)\right)$ .
