# Supplementary material for: Improving the stability of bivariate correlations using informative Bayesian priors: a Monte Carlo simulation study
Source: Front Psychol. 2023 Sep 7;14:1253452. doi: 10.3389/fpsyg.2023.1253452 (PMC10517051; doi:10.3389/fpsyg.2023.1253452)
Supplement: Supplementary file 1 [file Data_Sheet_1.PDF]

Supplementary Material

Improving the stability of bivariate correlations using informative Bayesian priors: A Monte Carlo simulation study

Carl Delfin[1,2*]

[1]Lund Clinical Research on Externalizing and Developmental Psychopathology (LU-CRED), Child and Adolescent Psychiatry, Department of Clinical Sciences Lund, Lund University, Lund, Sweden

[2]Centre for Ethics, Law and Mental Health (CELAM), Department of Psychiatry and Neurochemistry, Institute of Neuroscience and Physiology, Sahlgrenska Academy, University of Gothenburg, Gothenburg, Sweden

*** Correspondence:**
Carl Delfin
carl.delfin@med.lu.se

1 MCMC Diagnostics

No $\hat{R}$ value was above 1.00, indicating that all MCMC chains mixed well. Effective sample sizes (N_{EFF}) across all models ranged from 6121 to 22741, with a mean of 16575 and standard deviation of 1615. The Monte Carlo standard error (MCSE) across all models ranged from 0.000243 to 0.00364, with a mean of 0.0005536 and standard deviation of 0.0002879. There were no practical differences between the different models in terms of N_{EFF} (Table S1) or MCSE (Table S2). Five models (out of approximately 60 million) were indicated by CmdStan's diagnostic utility to have problems with either divergent transitions, transitions hitting maximum treedepth, or low E-BFMI values (see Betancourt, 2018 for details)[1]. All these models used a weakly informative prior with sample sizes between n = 11 and n = 13, for ρ = 0.2 (two models), ρ = 0.3 (two models), and ρ = 0.4 (one model). Since neither of these models exhibited deviant values of $\hat{R}$, N_{EFF}, or MCSE compared to remaining models, and since these models made up a negligible fraction of the total number of models, they were included in the final results.

[1] Betancourt, M. (2018). *A Conceptual Introduction to Hamiltonian Monte Carlo* (No. arXiv:1701.02434). arXiv. https://doi.org/10.48550/arXiv.1701.02434

2 Supplementary Figures and Tables

2.1 Supplementary Figures

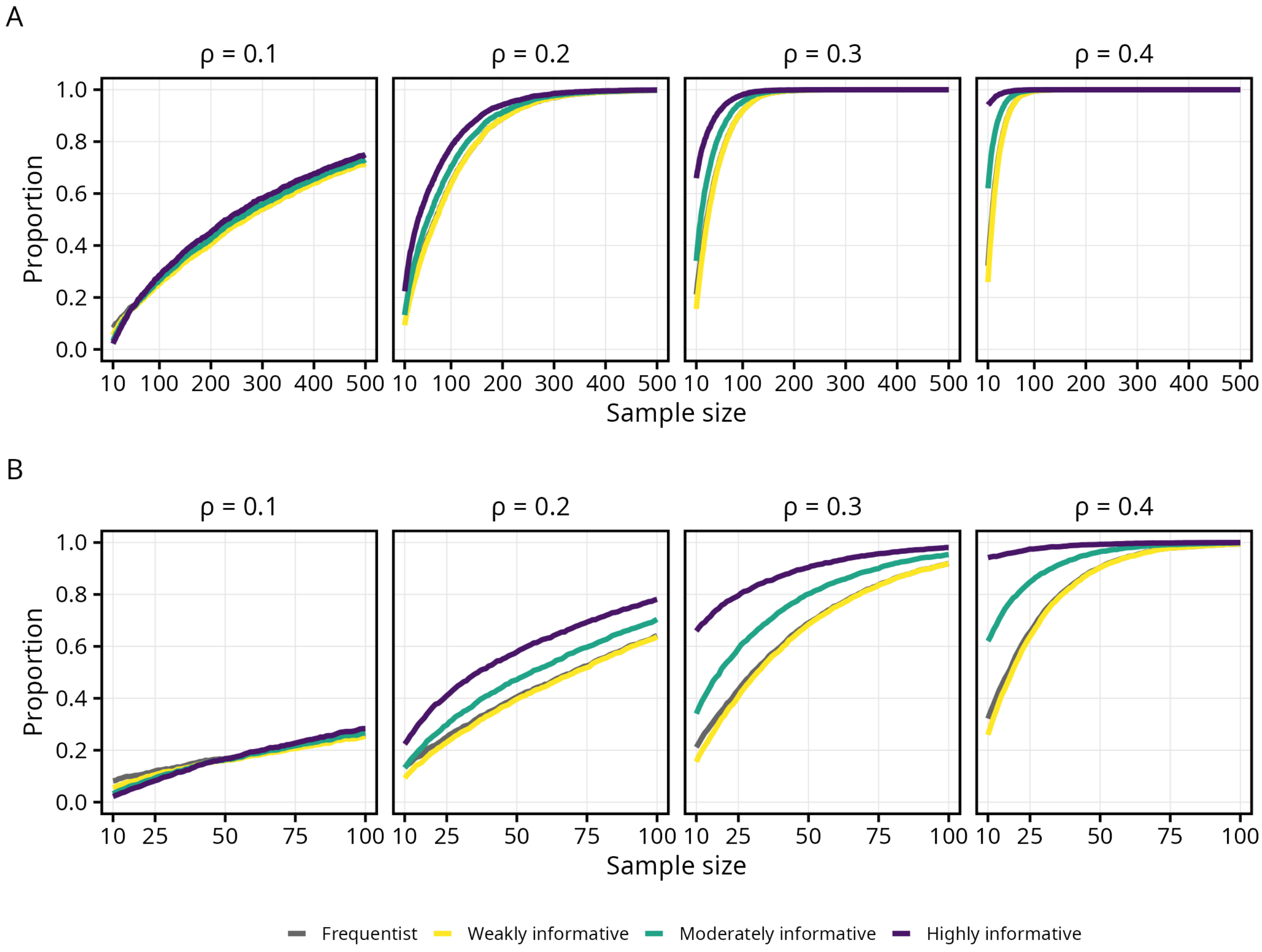

Supplementary Figure 1. Proportion of lower 90% interval bound above zero for each population correlation coefficient ρ. Lines represent the aggregated proportion across 10 000 replications for each model. (A) Results for all sample sizes. (B) Results up until N = 100.

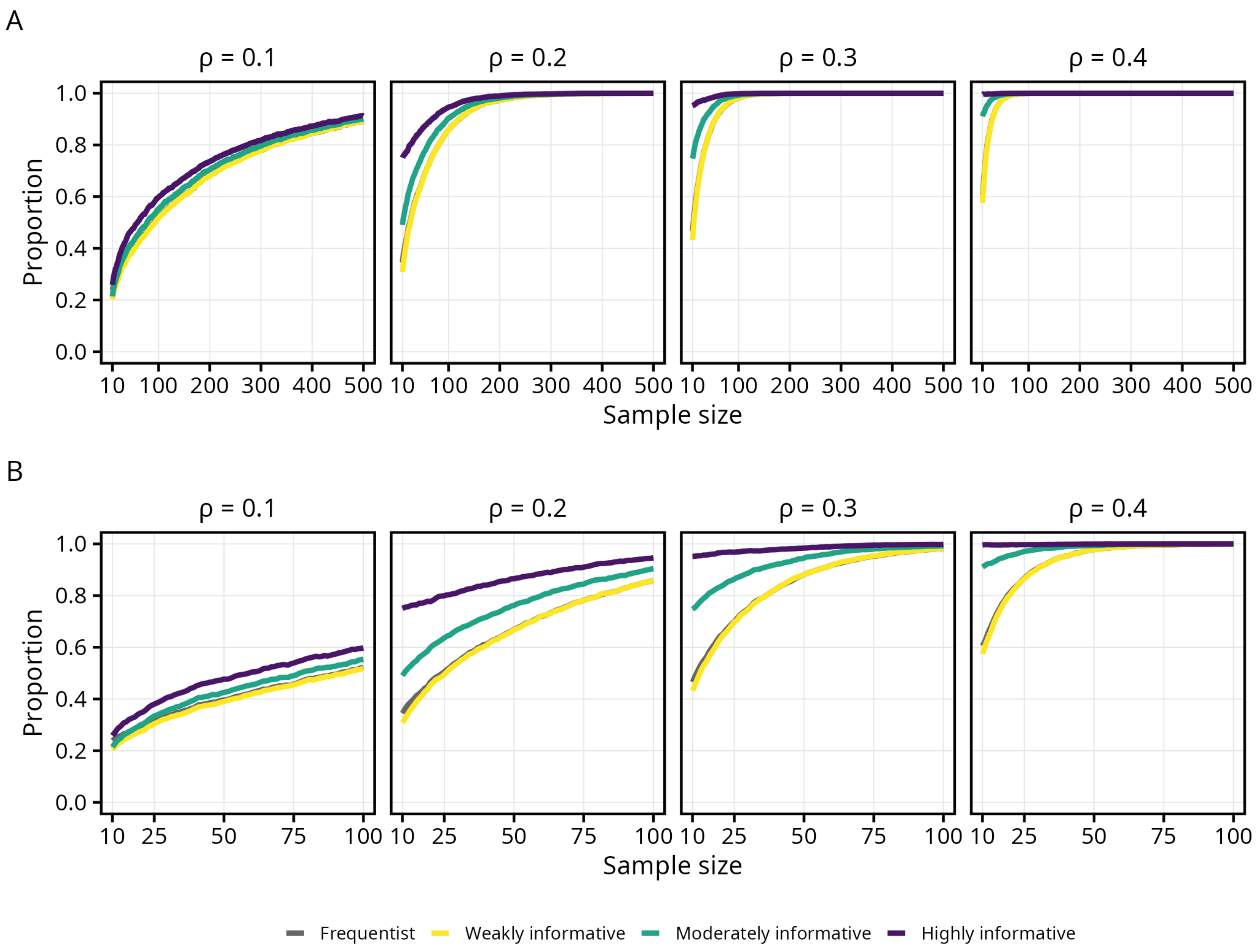

Supplementary Figure 2. Proportion of lower 66% interval bound above zero for each population correlation coefficient ρ. Lines represent the aggregated proportion across 10 000 replications for each model. (A) Results for all sample sizes. (B) Results up until N = 100.

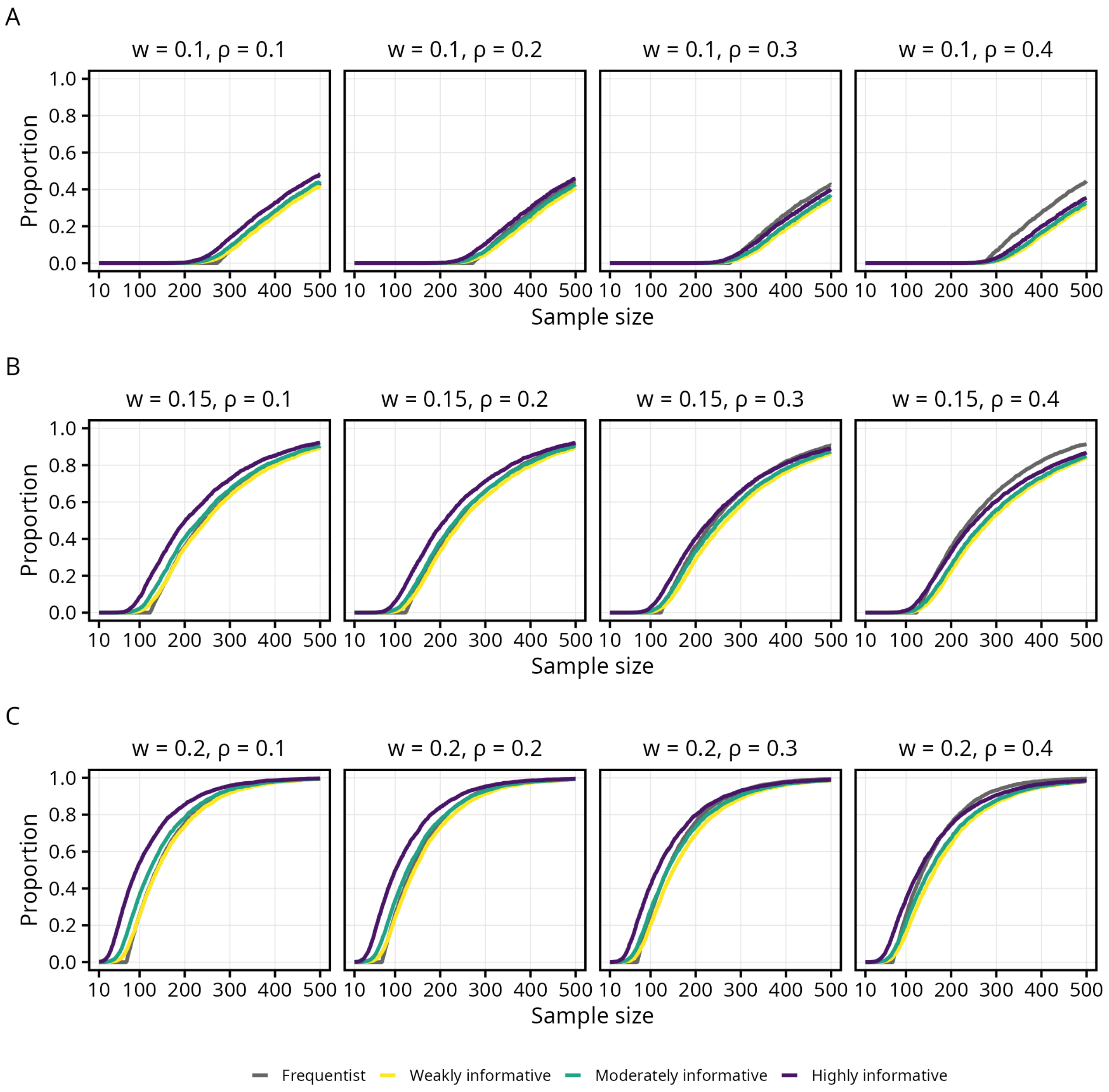

Supplementary Figure 3. Proportion of 90% intervals within different widths (w) of the Corridor of Stability (COS) for each population correlation coefficient ρ. Lines represent the aggregated proportion across 10 000 replications for each model. (A) $w = 0.1$. (B) $w = 0.15$. (C) $w = 0.2$.

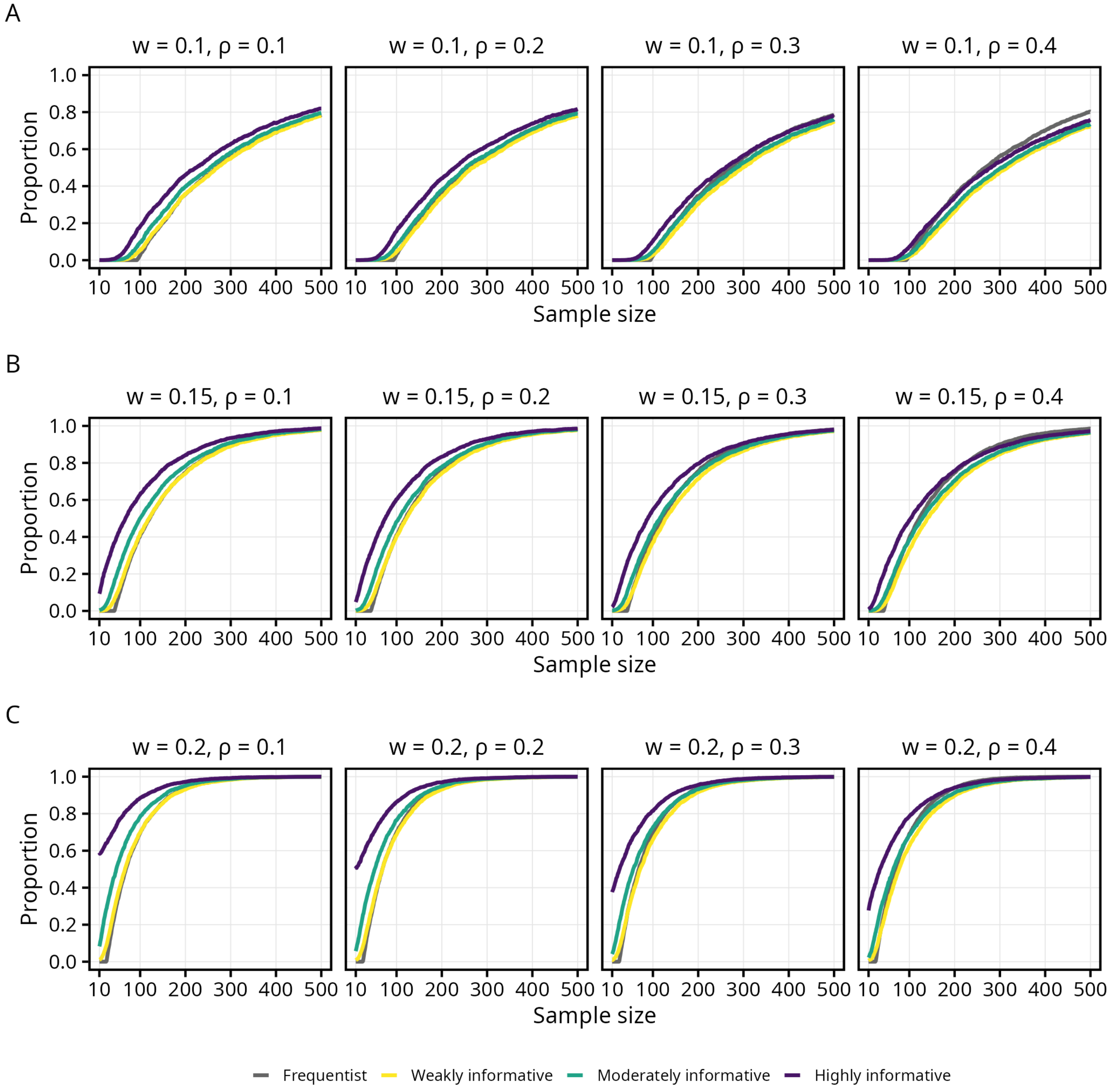

Supplementary Figure 4. Proportion of 66% intervals within different widths (w) of the Corridor of Stability (COS) for each population correlation coefficient ρ. Lines represent the aggregated proportion across 10 000 replications for each model. (A) $w = 0.1$. (B) $w = 0.15$. (C) $w = 0.2$.

2.2 Supplementary Tables

Table S1. Overview of effective sample size, for different ρ and models.

ρ	Model	Min	Max	Mean	SD
0.1	Highly informative	6361	22424	16874.98	1531.275
0.1	Moderately informative	6523	22336	16773.42	1552.553
0.1	Weakly informative	8432	22570	16603.64	1667.404
0.2	Highly informative	6121	22549	16813.09	1529.020
0.2	Moderately informative	6698	22469	16692.72	1557.639
0.2	Weakly informative	7370	22477	16518.22	1683.874
0.3	Highly informative	6891	22358	16695.12	1527.754
0.3	Moderately informative	6949	22575	16550.79	1567.734
0.3	Weakly informative	8058	22400	16367.51	1714.287
0.4	Highly informative	6600	22741	16531.60	1521.618
0.4	Moderately informative	6241	22313	16336.15	1587.833
0.4	Weakly informative	7157	22576	16147.89	1754.908

Table S2. Overview of Monte Carlo standard errors, for different ρ and models.

ρ	Model	Min	Max	Mean	SD
0.1	Highly informative	0.000258	0.00194	0.0005187628	0.0001971616
0.1	Moderately informative	0.000263	0.00243	0.0005644882	0.0002649450
0.1	Weakly informative	0.000266	0.00358	0.0006152460	0.0003741370
0.2	Highly informative	0.000254	0.00198	0.0005165367	0.0002006774
0.2	Moderately informative	0.000260	0.00275	0.0005605172	0.0002672481
0.2	Weakly informative	0.000257	0.00364	0.0006078517	0.0003702214
0.3	Highly informative	0.000253	0.00215	0.0005104090	0.0002045684
0.3	Moderately informative	0.000252	0.00261	0.0005509372	0.0002675333
0.3	Weakly informative	0.000253	0.00362	0.0005942517	0.0003629901
0.4	Highly informative	0.000244	0.00224	0.0004947676	0.0002012336
0.4	Moderately informative	0.000243	0.00279	0.0005354860	0.0002651518
0.4	Weakly informative	0.000247	0.00355	0.0005741066	0.0003519686

Table S3. Sample size required for obtaining a specific proportion (*P*) of estimates robustly different from zero, using a 90% interval, for different ρ and models. Percentage difference from the frequentist model is presented within parenthesis.

ρ	*P*	Model			
		Frequentist	Weakly informative	Moderately informative	Highly informative
0.1	0.80	> 500	> 500[a]	> 500[a]	> 500[a]
0.2	0.80	154	153 (-1%)	133 (-14%)	108 (-30%)
0.3	0.80	68	69 (+1%)	50 (-26%)	26 (-62%)
0.4	0.80	36	37 (+3%)	21 (-42%)	< 10 (-72%)[b]
0.1	0.90	> 500	> 500[a]	> 500[a]	> 500[a]
0.2	0.90	210	211[c]	186 (-11%)	159 (-24%)
0.3	0.90	94	94[c]	76 (-19%)	49 (-48%)
0.4	0.90	50	50[c]	33 (-34%)	10 (-80%)
0.1	0.95	> 500	> 500[a]	> 500[a]	> 500[a]
0.2	0.95	264	263[c]	244 (-8%)	215 (-19%)
0.3	0.95	117	117[c]	98 (-16%)	71 (-39%)
0.4	0.95	63	63[c]	45 (-29%)	15 (-76%)

Note. Robustly different from zero is defined here as the lower bound of the associated interval being above zero.

[a] Required sample size above 500 for all models; no percentage change calculated.

[b] Required sample size less than 10; numbers represent upper bound.

[c] Less than 1% difference from frequentist model; no percentage change calculated.

Table S4. Sample size required for obtaining a specific proportion (P) of estimates robustly different from zero, using a 66% interval, for different ρ and models. Percentage difference from the frequentist model is presented within parenthesis.

ρ	P	Model			
		Frequentist	Weakly informative	Moderately informative	Highly informative
0.1	0.80	324	324[c]	304 (-6%)	272 (-16%)
0.2	0.80	81	81[c]	60 (-26%)	26 (-68%)
0.3	0.80	37	37[c]	16 (-57%)	< 10 (-73%)[b]
0.4	0.80	19	20 (+5%)	< 10 (-47%)[b]	< 10 (-47%)[b]
0.1	0.90	> 500	> 500[a]	496 (-1%)	463 (-7%)
0.2	0.90	122	120 (-2%)	98 (-20%)	68 (-44%)
0.3	0.90	55	56 (+2%)	33 (-40%)	< 10 (-82%)[b]
0.4	0.90	29	30 (+3%)	10 (-66%)	< 10 (-66%)[b]
0.1	0.95	> 500	> 500[a]	> 500[a]	> 500[a]
0.2	0.95	158	158[c]	137 (-13%)	107 (-32%)
0.3	0.95	74	74[c]	52 (-30%)	10 (-86%)
0.4	0.95	39	39[c]	19 (-51%)	< 10 (-74%)[b]

Note. Robustly different from zero is defined here as the lower bound of the associated interval being above zero.

[a] Required sample size above 500 for all models; no percentage change calculated.

[b] Required sample size less than 10; numbers represent upper bound.

[c] Less than 1% difference from frequentist model; no percentage change calculated.

Table S5. Sample size required for obtaining a specific proportion (*P*) of 90% intervals within a specific width (*w*) of the Corridor of Stability, for different *ρ* and models. Percentage difference from the frequentist model is presented within parenthesis.

ρ	*P*	*w*	Model			
			Frequentist	Weakly informative	Moderately informative	Highly informative
0.1	0.80	0.20	218	226 (+4%)	206 (-6%)	172 (-21%)
0.2	0.80	0.20	214	229 (+7%)	213[a]	180 (-16%)
0.3	0.80	0.20	214	242 (+13%)	227 (+6%)	202 (-6%)
0.4	0.80	0.20	217	261 (+20%)	249 (+15%)	224 (+3%)
0.1	0.80	0.15	393	402 (+2%)	380 (-3%)	350 (-11%)
0.2	0.80	0.15	380	403 (+6%)	384 (+1%)	356 (-6%)
0.3	0.80	0.15	387	430 (+11%)	416 (+7%)	392 (+1%)
0.4	0.80	0.15	384	462 (+20%)	452 (+18%)	427 (+11%)
0.1	0.80	0.10	> 500	> 500[b]	> 500[b]	> 500[b]
0.2	0.80	0.10	> 500	> 500[b]	> 500[b]	> 500[b]
0.3	0.80	0.10	> 500	> 500[b]	> 500[b]	> 500[b]
0.4	0.80	0.10	> 500	> 500[b]	> 500[b]	> 500[b]
0.1	0.90	0.20	276	283 (+3%)	264 (-4%)	232 (-16%)
0.2	0.90	0.20	268	289 (+8%)	269[a]	240 (-10%)
0.3	0.90	0.20	274	310 (+13%)	295 (+8%)	266 (-3%)
0.4	0.90	0.20	269	332 (+23%)	324 (+20%)	294 (+9%)
0.1	0.90	0.15	499	> 500[a]	490 (-2%)	457 (-8%)
0.2	0.90	0.15	481	> 500 (+4%)[c]	495 (+3%)	460 (-4%)
0.3	0.90	0.15	488	> 500 (+2%)[c]	> 500 (+2%)[c]	> 500 (+2%)[c]
0.4	0.90	0.15	475	> 500 (+5%)[c]	> 500 (+5%)[c]	> 500 (+5%)[c]
0.1	0.90	0.10	> 500	> 500[b]	> 500[b]	> 500[b]
0.2	0.90	0.10	> 500	> 500[b]	> 500[b]	> 500[b]
0.3	0.90	0.10	> 500	> 500[b]	> 500[b]	> 500[b]
0.4	0.90	0.10	> 500	> 500[b]	> 500[b]	> 500[b]
0.1	0.95	0.20	333	346 (+4%)	325 (-2%)	289 (-13%)
0.2	0.95	0.20	323	343 (+6%)	328 (+2%)	297 (-8%)
0.3	0.95	0.20	330	378 (+15%)	363 (+10%)	336 (+2%)
0.4	0.95	0.20	323	402 (+24%)	391 (+21%)	363 (+12%)
0.1	0.95	0.15	> 500	> 500[b]	> 500[b]	> 500[b]
0.2	0.95	0.15	> 500	> 500[b]	> 500[b]	> 500[b]
0.3	0.95	0.15	> 500	> 500[b]	> 500[b]	> 500[b]
0.4	0.95	0.15	> 500	> 500[b]	> 500[b]	> 500[b]
0.1	0.95	0.10	> 500	> 500[b]	> 500[b]	> 500[b]
0.2	0.95	0.10	> 500	> 500[b]	> 500[b]	> 500[b]
0.3	0.95	0.10	> 500	> 500[b]	> 500[b]	> 500[b]
0.4	0.95	0.10	> 500	> 500[b]	> 500[b]	> 500[b]

[a] Less than 1% difference from frequentist model; no percentage change calculated.
[b] Required sample size above 500 for all models; no percentage change calculated.
[c] Required sample size above 500; numbers represent lower bound.

Table S6. Sample size required for obtaining a specific proportion (P) of 66% intervals within a specific width (w) of the Corridor of Stability, for different ρ and models. Percentage difference from the frequentist model is presented within parenthesis.

ρ	P	w	Model			
			Frequentist	Weakly informative	Moderately informative	Highly informative
0.1	0.80	0.20	129	128 (-1%)	106 (-18%)	65 (-50%)
0.2	0.80	0.20	126	131 (+4%)	112 (-11%)	77 (-39%)
0.3	0.80	0.20	127	139 (+9%)	123 (-3%)	94 (-26%)
0.4	0.80	0.20	128	148 (+16%)	136 (+6%)	107 (-16%)
0.1	0.80	0.15	230	230[a]	208 (-10%)	171 (-26%)
0.2	0.80	0.15	220	231 (+5%)	213 (-3%)	177 (-20%)
0.3	0.80	0.15	222	247 (+11%)	230 (+4%)	204 (-8%)
0.4	0.80	0.15	227	263 (+16%)	252 (+11%)	224 (-1%)
0.1	0.80	0.10	> 500	> 500[b]	> 500[b]	470 (-6%)
0.2	0.80	0.10	500	> 500[a]	> 500[a]	473 (-5%)
0.3	0.80	0.10	> 500	> 500[b]	> 500[b]	> 500[b]
0.4	0.80	0.10	496	> 500 (+1%)	> 500 (+1%)	> 500 (+1%)
0.1	0.90	0.20	169	172 (+2%)	153 (-9%)	111 (-34%)
0.2	0.90	0.20	164	174 (+6%)	156 (-5%)	122 (-26%)
0.3	0.90	0.20	172	189 (+10%)	174 (+1%)	140 (-19%)
0.4	0.90	0.20	170	202 (+19%)	191 (+12%)	161 (-5%)
0.1	0.90	0.15	308	313 (+2%)	289 (-6%)	254 (-18%)
0.2	0.90	0.15	295	312 (+6%)	293 (-1%)	260 (-12%)
0.3	0.90	0.15	308	336 (+9%)	320 (+4%)	291 (-6%)
0.4	0.90	0.15	299	360 (+20%)	346 (+16%)	319 (+7%)
0.1	0.90	0.10	> 500	> 500[b]	> 500[b]	> 500[b]
0.2	0.90	0.10	> 500	> 500[b]	> 500[b]	> 500[b]
0.3	0.90	0.10	> 500	> 500[b]	> 500[b]	> 500[b]
0.4	0.90	0.10	> 500	> 500[b]	> 500[b]	> 500[b]
0.1	0.95	0.20	218	220 (+1%)	199 (-9%)	158 (-28%)
0.2	0.95	0.20	209	220 (+5%)	204 (-2%)	165 (-21%)
0.3	0.95	0.20	218	238 (+9%)	223 (+2%)	193 (-11%)
0.4	0.95	0.20	212	258 (+22%)	244 (+15%)	213[a]
0.1	0.95	0.15	388	395 (+2%)	379 (-2%)	337 (-13%)
0.2	0.95	0.15	369	393 (+7%)	372 (+1%)	334 (-9%)
0.3	0.95	0.15	383	428 (+12%)	409 (+7%)	381 (-1%)
0.4	0.95	0.15	371	465 (+25%)	452 (+22%)	415 (+12%)
0.1	0.95	0.10	> 500	> 500[b]	> 500[b]	> 500[b]
0.2	0.95	0.10	> 500	> 500[b]	> 500[b]	> 500[b]
0.3	0.95	0.10	> 500	> 500[b]	> 500[b]	> 500[b]
0.4	0.95	0.10	> 500	> 500[b]	> 500[b]	> 500[b]

[a] Unknown difference from frequentist model; no percentage change calculated.
[b] Required sample size above 500 for all models; no percentage change calculated.
[c] Required sample size above 500; numbers represent lower bound.
